# Procedural Learning in Individuals with Amnestic Mild Cognitive Impairment and Alzheimer’s Dementia: a Systematic Review and Meta-analysis

**DOI:** 10.1007/s11065-020-09449-1

**Published:** 2020-09-08

**Authors:** Liselotte De Wit, Michael Marsiske, Deirdre O’Shea, Roy P.C. Kessels, Andrea M. Kurasz, Brittany DeFeis, Nancy Schaefer, Glenn E. Smith

**Affiliations:** 1 Department of Clinical and Health Psychology, University of Florida, P.O. Box 100165, Gainesville, FL 32610-0165 USA; 2 Donders Institute for Brain, Cognition and Behaviour, Radboud University, Montessorilaan 3, 6525 HR Nijmegen, The Netherlands; 3 Department of Medical Psychology & Radboudumc Alzheimer Center, Radboud University Medical Center, Postbus 9101, 6500 HB Nijmegen, The Netherlands; 4 University of Florida Health Science Center Libraries, University of Florida, SW Archer Rd, Gainesville, FL 32610 USA

**Keywords:** Alzheimer’s disease, Mild cognitive impairment, Procedural memory, Procedural learning, Pattern learning, Skill learning

## Abstract

The notion that procedural learning and memory is spared in Alzheimer’s disease (AD) has important implications for interventions aiming to build on intact cognitive functions. However, despite these clinical implications, there are mixed findings in the literature about whether or not procedural learning remains intact. This meta-analysis examines the standard mean difference of all published studies regarding procedural learning in AD dementia or amnestic Mild Cognitive Impairment (aMCI) compared to cognitively healthy older adults. Additionally, we conducted statistical equivalence analyses. Our systematic review showed that only a limited number of studies (*k* = 17) have compared procedural learning between individuals with aMCI or AD dementia and healthy controls. Our meta-analysis, which synthesized these studies, demonstrated that while procedural learning performance was not statistically equivalent between individuals with aMCI or AD dementia, and healthy older adults, the difference was clinically and statistically trivial. Although larger studies are needed, the present findings suggest that procedural learning does appear to remain spared in aMCI and AD dementia.

**Electronic supplementary material:**

The online version of this article (10.1007/s11065-020-09449-1) contains supplementary material, which is available to authorized users.

## Introduction

The notion that there are multiple kinds of memory systems that are supported by different brain areas was first studied in patient H.M., who became amnestic after bilateral removal of large parts of his medial temporal lobe (Milner, Corkin, & Teuber, 1968). Based on observations with H.M. and others, Squire (2004) offered an influential model of multiple memory systems. This model delineates two main memory systems: explicit memory and implicit memory. Explicit memory, also known as declarative memory, allows conscious recollection about facts and events (Squire, 2004), an ability that heavily relies on the medial temporal lobe (Eichenbaum & Lipton, 2008; van Strien, Cappaert, & Witter, 2009). In contrast, implicit memory is typically defined as a collection of abilities that are expressed through performance rather than recollection, for which conscious awareness is not required (Squire & Dede, 2015).

There are several subtypes of implicit memory. One sub-type of implicit memory is procedural memory, which encompasses skill learning (often motor skills), pattern learning, and habit learning, and is thought to mainly rely on the striatum and cerebellum, amongst other subcortical brain areas stages (Knowlton, Squire, Paulsen, Swerdlow, & Swenson, 1996; Sanes, Dimitrov, & Hallett, 1990; Seger, 2006). Skill learning tasks often involve an observable skill that improves with practice. Patient H.M., for instance, demonstrated improvement when presented with multiple trials on a motor skill task, without consciously remembering that he had ever performed the task before. Similarly, on coordinated tracing tasks, participants are asked to trace figures with a device (e.g. Hirono et al., 1997). Another commonly used visuomotor task is the rotary pursuit task, in which participants are required to track a target on a revolving wheel, trying to keep contact with a wand (e.g., Wright, 1999). Procedural memory tasks that have a more limited motor component include, for example, pattern learning tasks such as “prediction tasks,” or probabilistic classification tasks in which participants implicitly acquire cue-outcome associations (Baker, Bentham, & Kourtzi, 2015; Eldridge, Masterman, & Knowlton, 2002; Luft, Baker, Bentham, & Kourtzi, 2015) and the classic serial reaction time task (SRTT). In a SRTT for instance, participants are required to respond to visual cues that appear in different locations based on a specific sequence. While participants often have no awareness about this repeating sequence, a decrease in reaction times (RTs) is typically found as the sequences are repeated. Towards the end of the task, the embedded sequence changes, which often results in an increase in RTs. This increase in RTs is seen as a measure of learning of the embedded sequence. Nissen, and Bullemer (1987) who first described this task, showed that patients with anterograde amnesia due to Korsakoff demonstrated normal learning on this task while they had no awareness of the repeating pattern. Other types of procedural learning tasks with a limited motor component include tasks that require participants to solve novel puzzles, such as the such the Computerized Jigsaw Puzzle task (Hirono et al., 1997) or the Novel Task as part of the Rivermead Behavioural Memory Test-Third Edition (Wilson et al., 2008). Types of procedural memory tasks that rely on the perceptual systems rather than on the motor system include mirror reading, transformed script reading, and tactile character reading (Grober, Ausubel, Sliwinski, & Gordon, 1992; Hirono et al., 1997; Huberman, Moscovitch, & Freedman, 1994).

The finding that procedural memory remains intact in individuals with “pure” amnestic syndrome ultimately led to the emergence of studies testing procedural memory in other amnestic populations, such as individuals with dementia due to Alzheimer’s disease (AD). The medial temporal lobe is typically the first area to be affected in AD after which the pathology spreads to the posterior temporal lobes, parietal lobes, and frontal lobes, while brain areas that are thought to be important for procedural memory remain intact until the more severe stages (Sluimer et al., 2009; Whitwell, 2010). Correspondingly, declarative, or explicit, memory impairments are considered to an early hallmark of AD while implicit procedural memory and learning are thought to remain spared until the disease progresses. A recent activation likelihood estimation meta-analysis investigating the activation of brain areas in motor learning experiments (Hardwick, Rottschy, Miall, & Eickhoff, 2013) found activity across paradigms in the motor areas (left dorsal premotor cortex, bilateral supplementary motor cortex, the primary motor cortex), the primary sensory cortex, the left superior parietal lobule, the thalamus, the putamen and the cerebellum. When focusing on the SRTT specifically, results suggested that the bilateral dorsal premotor cortex, left basal ganglia, and thalamus were most consistently activated. In addition, the left caudate nucleus appeared of particular importance for implicit or unconscious versions of this task. The cerebellum was typically not activated during SRTTs. Regarding sensorimotor tasks, more consistent activations were found for the left dorsal premotor cortex and the bilateral cerebellum. The left basal ganglia, and thalamus are also typically activated during sensorimotor tasks (Hardwick, Rottschy, Miall, & Eickhoff, 2013). The neural correlates of perceptual procedural learning have, to our knowledge, have not been studied thus far.

To highlight the importance and clinical implications of procedural memory sparing, recent studies have described methods that aim to build on intact procedural learning in AD, (De Vreese et al., 2001; Greenaway et al., 2008; Harrison et al., 2007; Van Tilborg et al., 2007). For example, Van Halteren-Van Tilborg et al. (2007), described how intact procedural memory abilities can be used to reteach activities of daily living in patients with AD. However, there are several reasons why the conclusion that procedural learning remains intact in AD may be premature.

First, most studies that conclude that procedural learning remains spared in AD have done so based on the absence of a statistically significant difference when comparing procedural learning between individuals with AD and cognitively healthy older adults. However, the absence of a significant difference between groups can be explained by several determinants. The most obvious reason for the absence of an statistically significant different between groups is a lack of statistical power (i.e., sample sizes that are too small), resulting in Type II error. Reporting between-group effect sizes such as Cohen’s *d* or Hedges’ *g* may illuminate the role that small sample sizes can play in not reaching a statistical difference. To date, most articles that have examined procedural learning in AD and healthy older adults have not reported effect sizes (Baker et al., 2015; Desgranges et al., 1996; Eldridge et al., 2002; Gabrieli et al., 1993; Gobel et al., 2013; Grober et al., 1992; Hirono et al., 1997, 1996; Huberman et al., 1994; Kaemmerer, 2016; Knopman and Nissen, 1987, 1991; Luft et al., 2015; Merbah et al., 2011; Van Tilborg et al., 2011; Willingham et al., 1997; Wright, 1999). Alternatively, equivalence testing can be used to assess whether or not procedural memory functioning is statistically equivalent in aMCI/AD and healthy older adults (Lakens, 2017). To our knowledge, this type of analyses has not been used thus far to assess procedural memory sparing. Second, there are several studies that have found that procedural learning does not remain spared in AD (Grober et al., 1992; Merbah et al., 2011) or that have mixed or inconclusive results (Dick et al., 2001; Kaemmerer, 2016).

A potential contributing factor for inconclusive data regarding sparing of procedural learning in AD is the heterogeneity in disease severity between studies. AD is now understood as the (most common) etiology for a neurodegenerative process that first manifests as the MCI syndrome (Albert et al., 2011) and then often progresses to a full dementia syndrome, known as AD dementia (McKhann et al., 1984). The concept of Mild Cognitive Impairment (MCI) was formulated over 20 years ago (Petersen et al., 1999; Smith et al., 1996) as a target for research aiming to focus on individuals at risk for dementia. Per the current criteria, MCI is characterized by 1) a cognitive concern, 2) cognitive impairment on psychometric testing, 3) largely intact activities of daily living (ADLs), and 4) not meeting criteria for dementia (Albert et al., 2011). In MCI due to AD, explicit (declarative) memory is often the main cognitive domain to be affected (Petersen, 2004) This is often referred to as the amnestic subtype of MCI (aMCI). Explicit memory being the main cognitive domain to be impaired is concordant with the medial temporal lobe being the first brain area to be affected in AD. However, AD pathology is known to spread throughout the brain as the disease progresses, potentially affecting brain areas that are important for procedural learning such as the basal ganglia (Anderkova, Barton, & Rektorova, 2017). While procedural learning may remain spared in the MCI stage of AD, it may no longer be intact in the dementia stages of the disease. Still, studies of cognitive function in AD are generally constrained to individuals in the early (mild to moderate) stages of dementia. This may be because severe dementia patients are often not able to cooperate with even simple study procedures. Nevertheless, if AD severity explains the heterogeneity of findings regarding procedural learning in AD, one would expect to find more consistent sparing of procedural learning when examining pre-stages of AD dementia such as MCI.

As such, the aim of current meta-analyses was to address these issues of power and heterogeneity of disease severity. We aim to extend the previous literature by examining the standard mean difference of all studies that have been conducted thus far regarding procedural learning in AD dementia or aMCI compared to cognitively healthy older adults. If procedural learning remains spared in individuals with aMCI and AD, one would expect to find trivial standard mean differences when comparing procedural learning performances between these two patient groups and healthy older adults. The current meta-analysis addresses the power issues in previous studies by (1) calculating an effect size for each individual study that has been conducted on this topic that allows this comparison, (2) by providing a combined “meta” effect size based on all the individual studies, and (3) by conducting equivalence tests. To address the heterogeneity of disease severity, the difference in procedural learning between individuals with aMCI and healthy older adults as opposed to the difference between individuals with AD dementia and healthy older adults will also be explored.

## Methods

### Systematic Search

The PRISMA guidelines were followed for the literature search and selection procedures (Moher, Liberati, Tetzlaff, Altman, & PRISMA Group, 2009). An experienced librarian (NS) searched the MEDLINE and PsycInfo databases from inception to 09/09/2019. The search strategy included MeSH and PsycInfo controlled vocabulary terms and keywords and full-text language limits. The complete search strategy can be found in Supplement 1. A flow diagram with the numbers of the search is presented in Supplement 2, Figure S1.

#### Study Selection

Inclusion criteria were determined a priori and were assessed in the following order: (1) the article was in English or Dutch (as authors LDW and RCPK are fluent in both languages); the study used (2) at least one implicit memory/learning task that was completed by (3) a group of patients diagnosed with AD dementia or aMCI and (4) a control group of cognitively healthy older adults. Further inclusion criteria were that the article was (5) peer reviewed (dissertations were considered to be peer reviewed, because most committees consist of at least two experts on the topic) and (6) was original work (hence, review articles were excluded). The topic of the current meta-analysis was further narrowed by (7): the presence of an implicit memory task focused on *procedural* learning and (8) the statistics allowed comparison of procedural learning between the patient and control group, further described below. For articles that provided insufficient details to allow effect size calculation, the authors of the papers were contacted by email to ask for additional information. The response rate for this query was 24%. For those respondents, 100% indicated that additional data were no longer available as the data were/had been collected over 10 years ago. A flow diagram, depicting the inclusion and exclusion of articles can be found in Supplement 2. Two authors (LDW and DO) independently reviewed the list of potential articles produced by the search strategy.

### Data Synthesis

#### Procedural Learning Statistics and Effect Size Calculation

Outcome measures were reaction times (RTs), time to complete task, accuracy, errors, or statistics derived from these outcomes such as percentage correct. LDW calculated effect sizes (Cohen’s *d*, which were then converted to Hedges’ *g*) based on the *n* for both the patient and the control groups, along with one of the following sets of statistics:

1. Statistics on change scores, as calculated by subtracting the performance on the last exposure of a repeated sequence, last learning trial, or a familiar condition from the performance on a first exposure, new sequence, or unfamiliar condition. For studies that reported the mean change score within groups together with the *SD* (or *SE*) of the mean change score, Cohen’s *d* between groups was calculated as follows: $\frac{d=\left({M}_{ChangeControls}-{M}_{ChangeAD}\right)}{SD_{ChangePooled}}$
  *SD*_*ChangePooled*_ was calculated as follows: ${SD}_{ChangePooled}=\frac{\sqrt{{SD^2}_{ChangeControls\times nControls}+{SD^2}_{ChangeAD\times nAD}}}{n_{Controls}+{n}_{AD}}$When a *SE* was reported instead of a *SD*, *SE* was converted to *SD*.
  For studies that did not report within-group mean change scores, but reported the mean and *SD* (or *SE*) of both the performance on a repeated sequence, last learning trial, or the familiar condition as well as the performance on a first exposure, new sequence, or unfamiliar condition, unstandardized within-group mean change scores were calculated as follows: *M*_*Unfamiliar*_ − *M*_*Familiar*_
2. Of these within-group change scores, Cohen’s *ds* were calculated as described above. Instead of the pooled *SDs* or *SEs*, the *SDs* or *SEs* of the first exposure, new sequence, or unfamiliar condition were used in these studies (producing a maximally conservative estimates of effect size; this was done for six studies).For studies that analyzed a two-by-two interaction effect on condition (familiar versus unfamiliar condition by patient group versus control group), the *F-*statistic of the interaction effect was used to calculate Cohen’s *d*. For these studies, Cohen’s d was calculated as follows: $d=\sqrt{F\left({n}_{Controls}+{n}_{AD}\right)\times \left({n}_{Controls}\times {n}_{AD}\right)}$  

#### Data Extraction

Authors LDW, AMK, and BD extracted data from reading full texts. To ensure accuracy all statistics were independently reviewed by a second person of these tree data extractors. MM was consulted in case of doubt on which statistics to pull for the effect size calculation. Demographic information on the participant groups, including the *n*, age, percentage of male participants, and the diagnostic criteria used for the patient groups can be found in Table 1. The statistics used for the calculations and the calculated effect sizes are reported in Table 2.

**Table 1:** Demographic characteristics of the study samples

Authors	Year	Patient Group & Diagnostic Criteria	Mean Score Cognitive. Screener HC	Mean Score Cognitive screener MCI/ADD	HC *n*	MCI/ADD *n*	HC *M* age	MCI/ADD *M* age	HC %male	MCI/ADD % male
Pattern Learning Tasks										
AD Dementia										
Eldridge et al.	2002	Presence of Memory Disorder and ≥ 1 other cognitive Impairment	NR	MMSE: 22.4	10	8	81.2	81.2	30.0	62.5
Kaemmerer	2016	“Clinical Diagnosis,” criteria unspecified.	MMSE: 29.8	MMSE: 25.0	23	4	66.8	81.9	61.0	50.0
Knopman	1991	NINCDS-ADRDA	28.9	20.7	14	8	69.4	72.8	25.0	40.0
Knopman and Nissen	1987	NINCDS-ADRDA	NR	MMSE ≤25	13	28	68.5	71.7	61.5	45.7
Van Tilborg et al.	2011	NINCDS-ADRDA	MMSE: 27.6	MMSE: 20.6	10	10	74.3	82.8	50.0	40.0
Willingham et al.	1997	NINCSD-ADRDA	DRS: 136.7	DRS: 115.5	20	11	74.9	75	40.0	50.0
Amnestic Mild Cognitive Impairment or MCI likely due to AD										
Baker et al.	2015	NIAA + AD Etiology	NR	ACE-III: 86.5	11	11	67.7	69.8	71.4	71.4
Gobel et al.	2013	Petersen 2004; CDR 0–0.5; no ADL deficits	MMSE: 29.1	MMSE: 28.3	20	11	71.2	76.0	30.0	38.5
Luft et al.	2015	NIAA + AD Etiology	NR	ACE-III: 87.6	9	9	65.1	69.8	66.7	77.8
Other Procedural Learning Tasks										
AD Dementia										
Desgranges et al.	1996	McKhann et al. (1984)	NR	20.6	30	24	70.7	70.5	33.0	41.7
Gabrieli et al.	1993	McKhann et al. (1984)	NR	BDS:14.1	8	9	66.0	71.9	62.5	44.4
Grober et al.	1992	NINCSDS-ADRDA	BMS errors: 2.9	BMS errors: 13.5	18	16	76.9	83.4	NR	NR
Hirono et al.	1996	NINCDS-ADRDA	NR	CDR: 0.5 (*n* = 6), 1 (*n* = 33), and 2 (*n* = 8)	20	28	70.9	71.8	40.0	42.6
BCT TCR					14	31				
Hirono et al.	1997	NINCSDS-ADRDA	MMSE≥27	CDR: 0.5 and 1			68.0	68.3	47.4	45.5
BCT					19	3611				
CJP					19	11				
TCR					20	24				
Huberman et al.	1994	NINCSDS-ADRDA	DRS: 142.9	DRS: 124.4	23	7	67.5	74.7	NR	57.1
Merbah et al.	2011	NINCDS-ADRDA	MMSE: 28.9	MMSE: 23.2	30	29	74.9	74.9	36.7	36.7
Wright	1999	“Clinical diagnosis of AD,” criteria unspecified	MMSE: 29.4	MMSE: 25.6	12	12	77.4	76.7	92	47.4

*AD* Alzheimer’s disease, *ADD* Alzheimer’s Disease Dementia, *BCT* Bimanual Coordinated Tracing, *BMS* Blessed Mental Status, *BDS* Blessed Dementia Scale, *CJP* Computerized Jigsaw Puzzle, *HC* Healthy Controls, *M* Mean, *MMSE*, Mini-Mental State Exam, *MCI* Mild Cognitive Impairment, *NR* Not reported, *NINCDS-ADRDA* National Institute of Neurological and Communicative Diseases and Stroke/Alzheimer’s Disease and Related Disorders Association, *TCR* Tactile Character Reading

**Table 2:** Tasks, outcome measures and effect sized of the included studies

Authors	Year	Task	Outcome	*ES* calculated from:	Hedges’ *g*	*V*
Pattern Learning Tasks						
AD Dementia						
Kaemmerer (dissertation)	2016	SRTT	RT; Accuracy block 4 versus random block (of first-order sequences)	*M*_*change*_*, SD*_*pre*_*, N*	0.925	0.220
Van Tilborg et al.	2011	SRTT	RT; Accuracy	*F, N*	0.348	0.186
Eldridge et al.	2002	PT	%Correct	*M*_*change*_*, SE*_*pre*_*, N*	−0.903	0.227
Willingham et al.	1997	SRTT	RT; Accuracy	*F, N*	0.192	0.107
Knopman	1991	SRTT	Accuracy	*M*_*change*_*, SD*_*pre*_*, N*	−0.170	0.182
Knopman and Nissen	1987	SRTT	Median RT (ms) slope block 1–4; slope last repeated block vs non-repeating block	*M*_*change*_*, SD*_*change*_*, N*	−0.017	0.086
Amnestic Mild Cognitive Impairment or MCI due to AD						
Baker et al.	2015	PT	%Correct	*F, N*	−0.041	0.168
Luft et al.	2015	PT	Accuracy	*F, N*	0.135	0.021
Gobel et al.	2013	SRTT	%Correct	*M*_*change*_*, SE*_*change*_*, N*	−0.329	0.135
Other Procedural Learning Tasks						
AD Dementia						
Merbah et al.	2011	Mirror Reading	Mean reading time block 1–5	*F, N*	0.509	0.068
Wright et al. (dissertation)	1999	Rotary Pursuit	Mean time on target; block 1–5	*M*_*change*_*, SD*_*pre*_*, N*	0.026	0.117
Hirono et al.	1997	Computerized Jigsaw Puzzle; Tactile Character reading Bimanual Coordinated Tracing	Time to task completion pre divided by post-test (in *s*)	*F, N*	−0.015	0.067
Desgranges et al.	1996	Mirror Reading	Speed (in *sec*)	*M*_*change*_*, SD*_*pre*_	0.070	0.055
Hirono et al.	1996	Tactile character reading Bimanual Coordinated Tracing	Speed (in *s*)	*F, N*	0.531	0.071
Huberman et al.	1994	Transformed Script Reading	Speed (in *s*) Repeated vs. first reading & repeated vs non-repeated	*M*_*change*_*, SD*_*change*_*, N*	−0.049	0.134
Gabrieli et al.	1993	Mirror Tracing	Accuracy	*F, N*	−0.813	0.232
Grober et al.	1992	Mirror Reading	Block 1; Block 2. Speed (in *ms*)	*M*_*change*_*, SD*_*pre*_*, N*	−0.080	0.089

*AD* Alzheimer’s Disease, *F: F*-statistic of a two by two interaction effect between familiar vs. unfamiliar condition and patient group vs. HC, *M:* mean, *PT:* Prediction Task, *SD* Standard Deviation, *SD*_pre:_ Standard Deviation of the new/unstudied condition, *M*_*change:*_ mean change score, *SE:* Standard Error, *SE*_*change:*_ Standard Error of the change score, *SE*_pre:_ Standard Error of the new/unstudied condition, *SRTT:* Serial Reaction Time Task, *V:* Variance of Hedges’ *g*, *%Correct:* Percentage Correct. A negative Hedges’ *g* indicates that there was more procedural learning in aMCI/AD dementia patients than in healthy controls. This was true for 9 out of 17 studies

#### Hedges’ *g*

Separate between-group Cohen’s *d* effect sizes and effect size variances were derived using the Practical Meta-Analysis Effect Size Calculator by Wilson, retrieved in March 2019 (D. B. Wilson, n.d.). There was a large amount of variability in the types of procedural memory outcome measures that were used in the studies, including reaction and response time, error rates, accuracy rates and variables derived from these measures. Therefore, variables were reverse coded as required to ensure that in the current meta-analysis a negative effect size consistently indicates more procedural learning in aMCI/AD dementia patients than in healthy controls for all variables. Further, because Cohen’s *d* can be inflated in small sample sizes (Borenstein, Hedges, Higgins, & Rothstein, 2009), we converted Cohen’s *ds* to Hedges’ *gs*. Hedges’ *gs* were derived as follows: *g* = *d* × 1 - 3/[4 × (*df* – 1)]. The variance of Hedges’ *g* was calculated based on correction factor *J*. *J* is often calculated as follows: *J* = 1-(3/(4 × *df*-1), in which *df* is the residual degrees of freedom. For studies with more than one dependent variable (*DV)*, Hedges’ *g* effect sizes for the individual *DVs* were aggregated in a combined Hedges’ *g* effect size to avoid giving more weight to studies who listed more than one *DV* in the overall effect size of the meta-analysis. This was done using the aggregate function of the mAd package using R (Team, 2016). This method aggregates all within-study effect sizes by computing the unweighted mean of the dependent effect sizes (Borenstein, Hedges, Higgins, & Rothstein, 2011), which has been shown to be slightly favored over other methods for aggregating DVs (Hoyt, & Del Re, 2018).

### Analyses

#### Analytic Model

First, we fitted a meta-analytic model using a random effects model. Random effect models are preferred when heterogeneity is expected between studies based on, for instance, task, outcome measures, or participant samples (Borenstein et al., 2009). Due to the variability in types of procedural learning tasks and disease severity amongst the included studies, we calculated the overall effect size of the study by the use of a random-effects model. The heterogeneity tests were calculated with Metafor’s “rma” function for linear mixed effect model (Viechtbauer, 2010) run using R (Team, 2016). The rma function requires the input of the effect sizes of each individual study, the variance of each individual effect size, and the weights given to the study. For the current meta-analysis, we derived weights from the inverse of the variance terms for each individual effect size. The inverse variance method gives more weight to larger studies than to smaller studies, which helps minimize the imprecision of the pooled effect estimate (Borenstein et al., 2009).

#### A Priori Effect Size Boundaries and Equivalence Testing

A priori assumptions were made about which bounds of the standard mean difference (Hedges’ *g*) in procedural learning performance between groups are considered meaningful. Specifically, an absolute value of the standard mean difference of .200 is considered a small yet meaningful effect (Cohen, 1977). Therefore, for this meta-analysis, the bounds of a trivial or non-meaningful effect were set to a standard mean difference smaller than the absolute value of .200. Hence, if the Hedges’ *g* in procedural learning performance between healthy older adults compared to individuals with aMCI and AD dementia was smaller than the absolute value of .200, the effect was considered to be trivial. In addition, we conducted equivalence testing to assess if procedural memory functioning in individuals with aMCI and AD dementia is equivalent to procedural memory functioning in healthy older adults. We used the TOSTtwo function of the TOSTER package (Lakens, 2017) in R (Team, 2016), inputting the meta-effect size and total sample size, as well as the Hedges’ *g*s, and the sample sizes for the individual samples. Because we used the standard mean difference (Hedges’ *g*), a value of 1 was entered as the SD for each group.

#### Subgroup Analysis

In order to compare studies with AD dementia patient groups to studies with aMCI groups, we conducted subgroup-analysis. In random effects models, we assumed that the differences in results across studies are due variation between studies *that is greater than would be expected if the true effect was the same in all studies.* This is considered *true heterogeneity.* True heterogeneity can be further explored by subgroup/moderator analyses. In order to conduct sub-group analysis, we aimed to distinguish “true heterogeneity” from random error using a statistical test based on the *Q* statistic and *I*^*2*^ statistic (Borenstein et al., 2009). The *Q* statistic is a measure of weighted squared deviations (Borenstein et al., 2009) and the *I*^*2*^ indicates the proportion of between-studies variance in the total variance of the observed effect sizes (Borenstein et al., 2009).

#### Publication Bias

Publication bias is the phenomenon that studies with statistically significant findings get published more frequently. This potential bias in published studies can also bias meta-analyses (Borenstein et al., 2009). In the current meta-analysis, we assessed for publication bias in several ways. First, by the use of a funnel plot. A funnel plot is a way of depicting the relationship between the individual effect sizes of the studies that are included in a meta-analysis and their standard error. In a funnel plot, studies that have a smaller standard error (typically larger studies) tend to cluster around the mean effect size while studies with a larger standard error (often smaller studies) are displayed towards the bottom of the graph. Smaller studies often have more sampling error variation in effect sizes and their effect sizes tend to be spread out more. The “funnel” shape of the funnel plot depicts these differences (Borenstein et al., 2009). We also aimed to use the Rank Correlation Test for Funnel Plot Asymmetry. The Rank Correlation Test for Funnel Plot Asymmetry assesses the significant asymmetry of the effect sizes and their variance for the included studies. Last, the Rosenthal’s Fail-safe *N* provides a computation of how many missing studies there would have to be before the *p* value of the meta-analysis standard-mean difference became statistically nonsignificant (Borenstein et al., 2009). These tests to assess for publication bias were all ran using Metafor (Viechtbauer, 2010) in R (Team, 2016).

## Results

### Included Studies

The final study sample of the current meta-analysis consisted of 17 articles (*k* = 17; total *N* = 670): nine on pattern learning and eight on other types of procedural learning. Out of these nine articles on pattern learning, five studies compared healthy older adults to individuals with AD dementia (Eldridge et al., 2002; Gobel et al., 2013; Kaemmerer, 2016; Knopman and Nissen, 1991; Willingham et al., 1997) and three studies compared healthy older adults to individuals with aMCI (Baker et al., 2015; Luft et al., 2015; Van Tilborg et al., 2011). The other procedural learning articles all compared healthy older adults to individuals with AD dementia. The total number of individuals with AD dementia that completed the tasks in these studies was 296. The total number of individuals with amnestic MCI that completed the tasks in these studies was 29. The total number of healthy controls that completed the tasks in these studies was 343. The *n*s for each individual study can be found in Table 1.

### Overall Effect Size

**Fig. 1:**
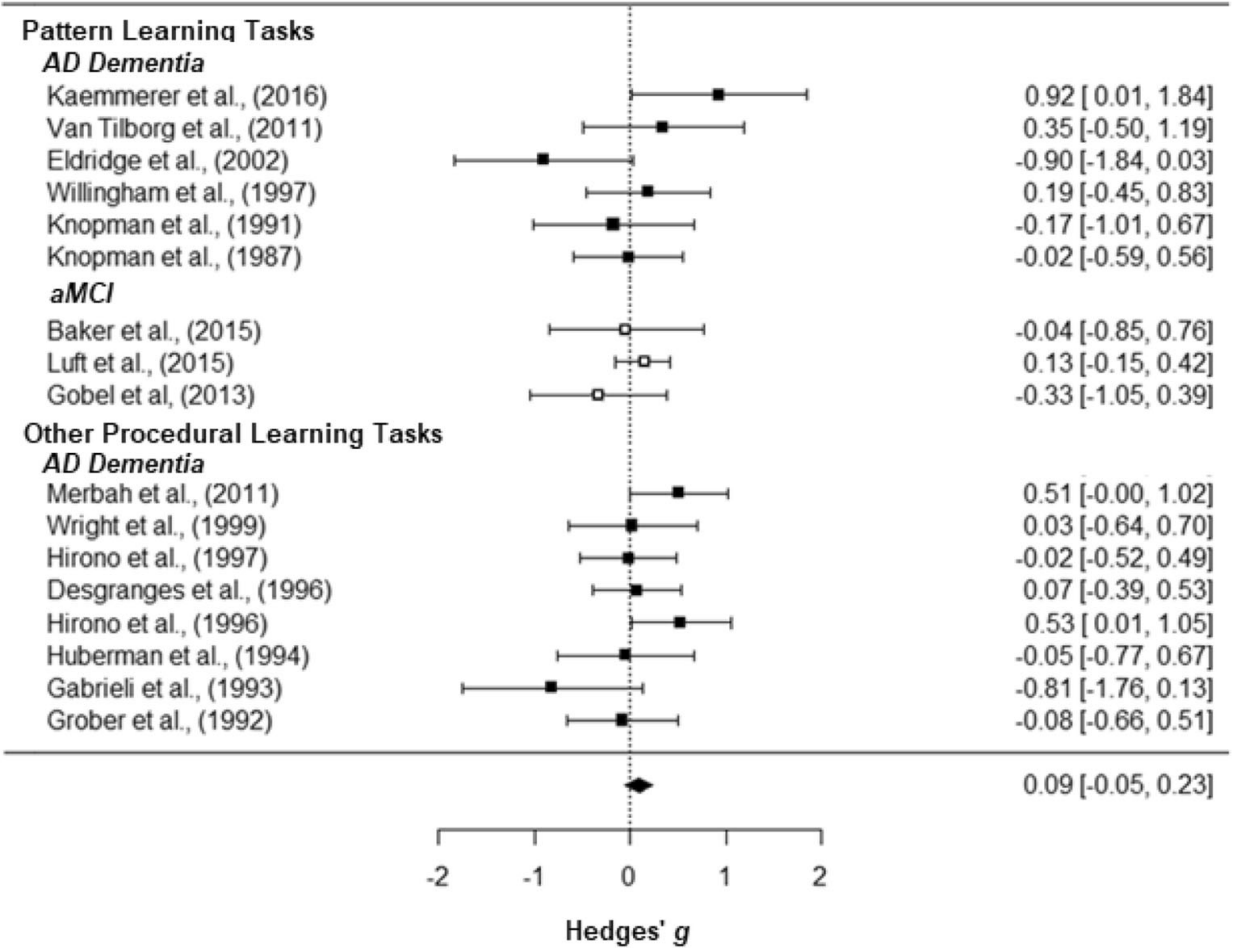
Forest plot. *Note*: Effect sizes of studies comparing HC and AD patients are depicted with black squares, and effect sizes of studies comparing HC and aMCI patients are depicted with white squares. Negative effect sizes indicate that there was more procedural learning in aMCI/AD patients than in healthy controls. This was found in nine out of 17 studies

The overall effect size of the current meta-analysis and the individual effect sizes of the included studies are reported in Table 2 and are depicted in the forest plot in Fig. 1. The overall effect size of the current meta-analysis was 0.092 (*SE* = 0.0751, 95% *CI* [−0. 048, 0.232]) and there was no statistically significant difference in procedural learning performance between individuals with aMCI/AD dementia and controls (*p* = .198).

**Fig. 2:**
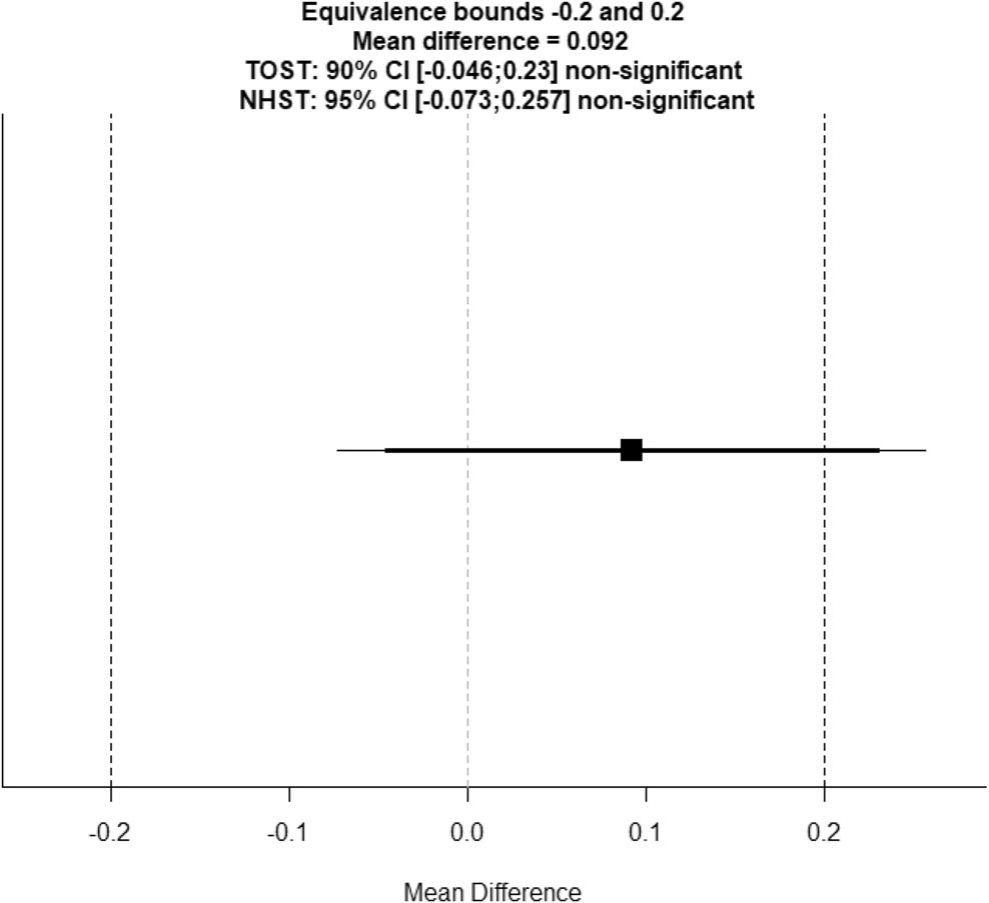
Equivalence plot

Based on the a-priori set bounds of a Hedges’ *g* (an absolute value of .200), the current results suggest that the difference in procedural learning performance between individuals with aMCI/dementia due to AD and healthy older adults can be considered trivial. However, as can also be seen in Fig. 2, the confidence interval of the overall effect sizes extends beyond the a-priori set equivalence bounds. Therefore, we cannot conclude that individuals with aMCI/AD have statistically *equivalent* procedural learning abilities when compared to cognitively healthy controls *t*(564.62) = −1.288, *p* = 0.099. To assess for equivalence more formally, we followed up by conducting statistical equivalence tests (Lakens, 2017). As we used a standard mean difference (Hedges’ *g*), a value of 1 was entered as the SD for each group. Based on these analyses, none of the 17 studies were found to have statistically equivalent results (*p* > .150).

We conducted a follow-up analysis with a subset of studies that had similar *DVs*, to assess whether the aggregation of different *DVs* biased the results. Specifically, in this analysis we only included studies that assessed speed (i.e., reaction time or time of task completion) and excluded all effect sizes that assessed accuracy. This analysis yielded an overall effect size of 0.134 (*SE* = 0.102, 95% *CI* [−0.0665, 0.3347], *p* = 0.190), demonstrating that also with a smaller subset of more similar outcome measures, there was no statistically significant difference in procedural learning performance between the two groups.

### Effect Sizes of the MCI and AD Subgroups

The *Q-*statistic was not statistically significant (*Q*(16) = 19.461, *p* = .246, *I*^*2*^ = 0.00). We conducted a subgroup analysis to assess the effect of group (aMCI vs AD as compared to healthy controls). Our results indicated that group was not statistically significant (*QM* (1) = 0.113 (*p* = .737). However, to gauge the overall effect size for the studies that included individuals with aMCI as opposed to studies that included individuals with AD dementia, an overall effect size for these studies was calculated. The overall effect size for all studies that compared patients with aMCI to healthy controls was 0.062 (*SE* = 0.127, 95% *CI* [−0.187, 0.312], *p* = 0.626). The overall effect size for all studies that compared patients with AD dementia to healthy controls was 0.106 (*SE* = 0.093, 95% *CI* [−0.076, 0.288], *p* = 0.160.

#### Publication Bias

**Fig. 3:**
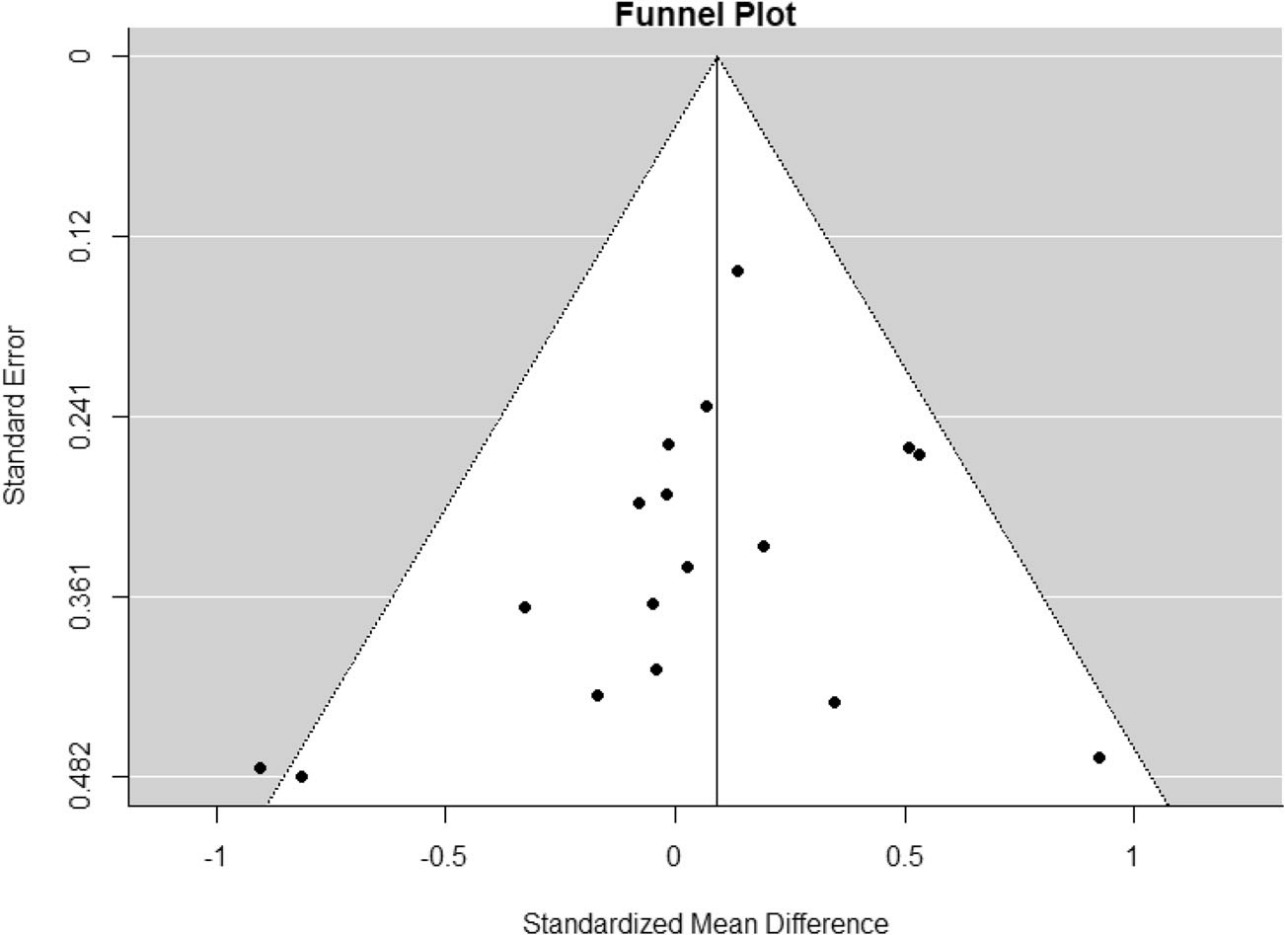
Funnel Plot showing the relationship between the individual effect sizes per study and their standard error

The funnel plot, which was not indicative of publication bias, is depicted in Figure 3. To statistically test if publication bias plays a significant role in the current meta-analysis, the Rank Correlation Test for Funnel Plot Asymmetry was conducted. As expected, the Rank Correlation Test for Funnel Plot Asymmetry was not statistically significant (Kendall’s tau = −0.105, *p* = 0.457), meaning that there was no statistically significant asymmetry in the Hedges’ *g* and the variance for the included studies. Hence, no evidence of publication bias was found. Given that there was no statistical significance between the groups, Rosenthal’s Fail-safe *N* was 0.

## Discussion

The medial temporal lobe is typically the first area to be affected in AD, while brain areas important for procedural memory, such as the basal ganglia and the cerebellum, remain intact until the more severe stages (Sluimer et al., 2009; Whitwell, 2010). Correspondingly, declarative learning and memory are considered a hallmark criterion of AD, while procedural learning are thought to remain intact until the disease progresses. The present meta-analysis is the first quantitative summary of procedural learning in AD. In this meta-analysis, we examined the standard mean difference of all studies that have been conducted thus far regarding procedural learning in AD dementia or aMCI (presumably due to AD) when compared to cognitively healthy older adults. Our findings indicate that the difference in procedural learning in individuals with aMCI or AD dementia compared to cognitively healthy older adults was not statistically significant and smaller than the a-priori set bounds for a trivial effect. This trivial difference between healthy controls and individuals with aMCI/AD dementia was found across different types of tasks including pattern learning and other procedural learning tasks. However, follow-up equivalence testing demonstrated that the findings were also not statistically equivalent between both groups.

### The Role of AD Severity

Only three of the 17 included studies contained an aMCI patient group. This small number of studies with an aMCI group yielded a small sample of individuals with aMCI (*N* = 29). Thus, the power for our subgroup analysis examining the difference in procedural learning in aMCI and healthy older adults as compared to the difference between AD dementia and healthy older adults was low and, unsurprisingly, not statistically significant. Therefore, no strong conclusions can be drawn regarding the effect of AD disease severity (the dementia versus the MCI phase) on procedural learning. Qualitatively, however, the effect size of studies comparing patients with aMCI to healthy controls appeared smaller than the effect size of studies comparing patients with AD dementia to healthy controls. Specifically, the individual effect sizes for the studies comparing procedural learning between healthy controls and aMCI were either trivial (Luft et al., 2015) or even negative (Baker et al., 2015; Gobel et al., 2013). This finding suggests that procedural learning appears to remain spared in aMCI. An additional consideration with regard to AD severity is the difficulty of testing individuals with severe AD dementia. While procedural learning may remain intact in these individuals, the ability to measure procedural learning can be confounded by variables such as the ability to understand and remember test instructions. As such, dementia patients with impairments in language comprehension or working memory may not be able to complete procedural learning tasks while they still may be able to learn new specific skills.

### Additional Factors of Consideration and Limitations

In the majority of the studies included in the current meta-analysis, a subgroup of participants were excluded from analyses, for instance because they were unable to perform the task (Baker et al., 2015; Gobel et al., 2013; Hirono et al., 1996; Kaemmerer, 2016; Knopman, 1991; Knopman & Nissen, 1987; Luft et al., 2015; Merbah et al., 2011; Willingham et al., 1997). This could represent a methodological error, analogous to excluding delayed recall scores of 0 from studies of declarative memory in AD. For future studies, authors should consider the problem of data missing not at random, i.e., specifically include the biases generated if the dependent variable (e.g. dementia severity) associates with missingness. To assess for the potential extent of this bias, we recommend reporting how many participants were able to complete screening tasks but unable to complete the procedural memory tasks. Furthermore, exploring predictors of participants who were able to complete the tasks versus those who were unable to complete the tasks is relevant to assess if individuals who were unable to complete the tasks were more cognitively impaired. In any event, the effect size in the current meta-analysis may be dampened as a result of studies excluding individuals that were unable, i.e. scoring at the ‘floor’ or unable to finish, the procedural memory tasks.

### Clinical Implications

Procedural learning in AD dementia and aMCI has important clinical implications (De Vreese et al., 2001; Greenaway et al., 2008; Harrison et al., 2007; Van Tilborg et al., 2007), for instance, for studies aiming to build on procedural memory to help compensate for memory loss (Greenaway et al., 2008; Chandler et al., 2019). Our findings suggest that in combined analysis comparing AD dementia and aMCI samples to health controls, procedural learning differences are statistically and clinically trivial, and thereby suggest no contra-indications for the use of procedural memory in efforts to compensate for areas of impaired declarative memory.

### Future Directions

It is theoretically and clinically important to help determine that procedural memory in MCI and mild dementia is equivalent to healthy controls. However, greater statistical power is needed for equivalence testing than for more traditional testing of differences. Thus, we recommend that large scale studies be conducted to enable stronger conclusions about statistical equivalence in procedural learning between healthy controls and individuals with aMCI/AD. For future studies with small sample sizes, reporting effect sizes is crucial for quantifying differences between groups. The best way to capture changes in procedural learning throughout the course of AD, of course, is by following healthy older adults over time. Future studies should therefore undertake longitudinal analyses. Last, none of the studies included in this meta-analysis included imaging or AD biomarkers. Given the rapidly developing changes in AD research criteria (e.g., Jack et al., 2018), AD biomarkers such as APOE-4 status and amyloid/tau positivity status should also be explored in relation to procedural learning.

### Conclusion

The present meta-analysis is the first quantitative summary of the literature on procedural learning performance in aMCI and AD dementia. It offers insight into whether procedural learning remains spared in the early phases of progressive cognitive decline. Thus far, only a small number of studies (*k* = 17) have compared procedural learning between healthy controls and individuals with aMCI/AD, totaling a small total number of participants (albeit with a total *N* of 296 healthy controls and 274 patients). Our small total sample, which affected our power and our resulting ability to find equivalence between groups, remains a significant limitation. However, our finding that the overall standard mean difference in procedural learning performance in individuals with aMCI or AD dementia compared to healthy older adults is both clinically and statistically trivial suggests that procedural learning appears to remain intact in aMCI and AD dementia phases.

## Electronic supplementary material

ESM 1 (DOCX 38.6 kb)
