# Supplementary material for: Procedural Learning in Individuals with Amnestic Mild Cognitive Impairment and Alzheimer’s Dementia: a Systematic Review and Meta-analysis
Source: Neuropsychol Rev. 2020 Sep 8;31(1):103–14. doi: 10.1007/s11065-020-09449-1 (PMC7889687; doi:10.1007/s11065-020-09449-1)
Supplement: Supplementary file 1 — (DOCX 38.6 kb) [file 11065_2020_9449_MOESM1_ESM.docx]

**Supplemental Materials**

**Supplement 1. Search Strategy conducted on 09/09/2019.**

**PubMed:** (((((("mild cognitive impairment"[tiab] OR "mild cognitive impairments" [tiab] OR "MCI"[tiab] OR "mild neurocognitive disorder"[tiab] OR "mild neurocognitive disorders"[tiab] OR "mild neurocognitive impairment"[tiab]) AND ("Amnesia"[Mesh] OR amnesi*[tiab] OR "Memory"[Mesh:NoExp] OR memory[tiab])) OR ("Alzheimer Disease"[Mesh] OR Alzheimer*[tiab] OR "AD"[tiab])) AND ("Repetition Priming"[Mesh] OR "procedural memory"[tiab] OR "instrumental memory" [tiab] OR "motor memory" [tiab] OR "motor-skill memory" [tiab] OR "muscle memory" [tiab] OR "nondeclarative memory" [tiab] OR "non-declarative memory" [tiab] OR "sequence memory" [tiab] OR "implicit memory" [tiab] OR "habit memory" [tiab] OR "skill memory" [tiab] OR "unconscious memory" [tiab] OR "pattern memory" [tiab] OR "spatial memory" [tiab] OR "spatial memory” [tiab] OR “Spatial memory”[mh] OR “visuospatial memory”[tiab] OR “visuo-spatial memory” [tiab] OR "visuospatial learning" [tiab] OR "visuo-spatial learning" [tiab] OR "procedural learning" [tiab] OR "instrumental learning" [tiab] OR "motor learning" [tiab] OR "motor-skill learning" [tiab] OR ((muscle[tiab] OR muscles[tiab]) AND (learning[tiab] OR memory[tiab])) OR ("Muscles"[Mesh] AND "Learning"[Mesh]) OR "nondeclarative learning" [tiab] OR "non-declarative learning" [tiab] OR "sequence learning" [tiab] OR "implicit learning" [tiab] OR "habit learning" [tiab] OR "skill learning" [tiab] OR "unconscious learning" [tiab] OR "pattern learning" [tiab] OR priming[tiab] OR "skill learning" [tiab] OR "unconscious memory" [tiab] OR "pattern memory" [tiab] OR "spatial memory" [tiab] OR "spatial learning" [tiab] OR “visuospatial memory”[tiab] OR “visuo-spatial memory” [tiab] OR "visuospatial learning" [tiab] OR "visuo-spatial learning" [tiab])))) NOT "review"[Publication Type] Filters: Humans; Dutch; English

**PsycINFO:** ((((DE "Cognitive Impairment" AND (TI mild OR AB mild)) OR (TI "mild cognitive impairment*" OR "MCI" OR "mild neurocognitive disorder*" OR "mild neurocognitive impairment*" OR "mild neuro-cognitive impairment*") OR (AB "mild cognitive impairment*" OR "MCI" OR "mild neurocognitive disorder*" OR "mild neurocognitive impairment*" OR "mild neuro-cognitive impairment*")) AND (DE "Amnesia" OR DE "Memory Disorders" OR DE "Anterograde Amnesia" OR DE "Global Amnesia" OR DE "Retrograde Amnesia" OR (TI amnes* OR memory) OR (AB amnes* OR memory))) OR (DE "Alzheimer's Disease" OR (TI alzheimer* OR “AD”) OR (AB alzheimer* OR “AD”))) AND ((DE "Priming" OR DE "Implicit Memory" OR DE "Visuospatial Memory" OR DE "Spatial Memory") OR (TI "procedural memory" OR "instrumental memory" OR "motor memory" OR "motor-skill memory" OR "muscle memory" OR "nondeclarative memory" OR "non-declarative memory" OR "sequence memory" OR "implicit memory" OR "habit memory" OR "skill memory" OR "unconscious memory" OR "pattern memory" OR "spatial memory" OR "procedural learning" OR "instrumental learning" OR "motor learning" OR "motor-skill learning” OR “muscle learning” OR "nondeclarative learning" OR "non-declarative learning" OR "sequence learning" OR "implicit learning" OR "habit learning" OR "skill learning” OR "unconscious learning" OR "pattern learning" OR priming OR "Implicit memory" OR “visuospatial learning” OR “visuo-spatial learning” OR “visuospatial memory” OR “visuo-spatial learning”) OR (AB "procedural memory" OR "instrumental memory" OR "motor memory" OR "motor-skill memory" OR "muscle memory" OR "nondeclarative memory" OR "non-declarative memory" OR "sequence memory" OR "implicit memory" OR "habit memory" OR "skill memory" OR "unconscious memory" OR "pattern memory" OR "spatial memory" OR "procedural learning" OR "instrumental learning" OR "motor learning" OR "motor-skill learning” OR “muscle learning” OR "nondeclarative learning" OR "non-declarative learning" OR "sequence learning" OR "implicit learning" OR "habit learning" OR "skill learning" OR "unconscious learning" OR "pattern learning" OR priming OR "Implicit learning" OR “visuospatial learning” OR “visuo-spatial learning” OR “visuospatial memory” OR “visuo-spatial learning”)). Limiters: Publication Type: All Journals, Dissertation Abstract; Language: Dutch, English, Population Group: Human; Methodology: Brain Imaging, Clinical Case Study, Clinical Trial, Empirical Study, Experimental Replication; Follow-up Study; Longitudinal Study, Prospective Study; Retrospective Study; Field Study, Interview. Focus Group Mathematical Model, Non-clinical Case Study, Qualitative Study, Quantitative Study Scientific Simulation. Treatment Outcome, Twin Study

**Supplement 2. Flow Diagram (adapted from PRISMA 2009).**

Full-text articles assessed for eligibility:
*n* = 274

Records identified through database searching (search conducted on 09/09/2019)
Total *n* = 1599
PubMed *n* = 861
PsycInfo *n* = 738

Records excluded
*n* = 1111

Records screened based on title and abstract
*n* = 1385

Records after removing duplicates
*n* =1385

Articles excluded based on full texts for the following reasons (total *n* = 257):
No Alzheimer’s or amnestic MCI: *n* = 7
No appropriate reference group: *n* = 6
No Implicit Memory Task: *n* = 20
No Original Work: *n* = 6
No Procedural Memory Task: *n* = 193 Insufficient information to calculate ESs: *n* = 25

Studies included in quantitative synthesis (meta-analysis)
*n* = 17
